# A systematic review and meta-analysis of effect of leucocyte- and platelet-rich fibrin on dental extraction

**DOI:** 10.4317/medoral.26724

**Published:** 2024-10-13

**Authors:** Xu Wang, Fu Xin, Siwei Zhou

**Affiliations:** 1 Department of Stomatology, Shijiazhuang Fourth Hospital, Shijiazhuang City, Hebei Province, China

## Abstract

**Background:**

Dental extraction is the most common oral surgery, but it leads to the remodelling of the socket, such that an implant is required for repair. We performed meta-analysis to determine whether leucocyte- and platelet-rich fibrin (L-PRF) improves dental extraction.

**Material and Methods:**

Following a search of Scopus, Web of science, ProQuest and PubMed, six relevant studies were included (239 patients treated with L-PRF after dental extraction).

**Results:**

The results provide higher percentage of bone formation after dental extraction in L-PRF implant patients with a mean difference of -13.16 (-15.89, -10.43) than control. Socket filling and horizontal width were also higher in the L-PRF implant group. A sub-group meta-analysis showed a significantly higher healing index 7 and 14 days after dental extraction in the L-PRF-treated group. The VAS score for pain stimuli was lower in the L-PRF group with a mean difference of 1.26 (1.00, 1.51) than control group; the difference in the heterogeneity of the studies was significant.

**Conclusions:**

These results show that L-PRF prevents ridge formation by improving the percentage of bone formation and socket width (improved horizontal width and socket filling). In such patients, the healing index was higher and the VAS score for pain stimuli lower than in the control group.

** Key words:**Dental extraction, L-PRF, socket filling, healing index.

## Introduction

Following tooth extraction, the socket undergoes remodelling such that repair treatment requires an implant (1). Clinical studies that have examined the changes in the soft and hard tissues after tooth extraction have reported a reduction of bone (2) and thus a delay in the healing process (3). Post-extraction bone loss includes a reduction of the blood supply (4) and is aggravated by factors such as systemic diseases, smoking status, flap elevation, morphology of the socket and extraction of the neighbouring teeth (5). Pain intensity and healing, and thus the adequate use of analgesic drugs, are also important factors in ridge prevention following tooth extraction (6). However, while several techniques have been used to reduce pain, healing time and loss of bone after tooth extraction (7), none are particularly effective, either clinically or with respect to their cost.

L-PRF, derived from platelet concentrate and leucocytes, has been used to stimulate bone growth (8). This second-generation preparation is free of the limitations of the first-generation product and is both inexpensive and easy to obtain, without the need for biochemical handling. L-PRF induces protein synthesis and growth factor release, and promotes rapid cicatricial tissue remodelling, wound healing, cell proliferation and neovascularisation (9). However, its effects on bone formation is unclear (10); both ridge prevention and no effect on bone loss have been reported (11). Therefore, here, we performed systematic review and meta-analysis to determine effect of L-PRF on dental extraction.

## Material and Methods

This study follows the guideline of the Preferred Reporting Items for Systematic Reviews and Meta-Analyses (PRISMA) for conducting and reporting meta-analyses and systematic reviews. The protocol of this study was registered with PROSPERO (CRD42022357723).

- Search Strategy

Databases of Scopus, Web of Science, ProQuest and PubMed were used to do detailed literature search. The following broad terms were searched with combinations of keywords: ridge prevention, healing index, oral implant with L-PRF.

All records from different databases were exported to Excel in CSV file format. After all four CSV file records from the different databases had been compiled, duplicate records were removed. All remaining records were screened by examining the titles and Abstracts of each one and removing insignificant records based on the criteria of inclusion and exclusion of our study. The remaining records were subjected to a full-text review before their inclusion in the analysis.

- Data extraction and risk of bias

Informations like first author, year of publication, country name, number of patients, mean age, proportion of males and females, socket width (L-PRF/control), healing index (L-PRF/control), VAS (L-PRF/control), conclusion and references were extracted from all the selected articles (12-17).

The risk of bias were assessed by 2 reviewers from all included studies, using Cochrane Collaboration RoB 2. A randomised controlled trial (RCT) checklist was used to evaluate all of the included studies as per directions. If all criteria were met, the study was rated as having a low risk of bias considered when all the criteria met; risk of bias considered high, in case of one or more criteria didn’t meet; unclear considered based on unclear domain more than one. Moreover third reviewer consult, if there is disagreement between the first two reviewer.

- Selection criteria

Studies with the following features were included: comparison of ridge prevention, i.e., socket dimension, healing index and Visual Analogue Scale (VAS); randomised clinical trial including split-mouth studies; clinical studies. Studies with the following features were excluded: comparison of pain sensation using different scales; patients with comorbidities; pre-clinical studies. In all, on inclusion criteria 239 articles were excluded. Ultimately, six full text articles were included in the meta-analysis was performed on six full test.

- Statistical analysis

Results are represented as means±SD. A statistical evaluation indicated significant heterogeneity in effect size, and thus a random effects model was used. Differences in outcome between controls and an L-PRF group were represented using a forest plot, and a Q test and I2 index were used to assess statistical heterogeneity, expressed as a percentage of the variation (where I2=75% was high heterogeneity; I2=50% was moderate; I2=25% was low). Subgroup analyses were performed to determine the effect of L-PRF on socket width (horizontal width, vertical width, and socket fill) and the healing index (post-operative days 7 and 14) after dental extraction. Review Manager (RevMan 5.3. Copenhagen: The Nordic Cochrane Centre, The Cochrane Collaboration, 2014) was used to perform statistical analysis.

## Results

**Figure 1:**
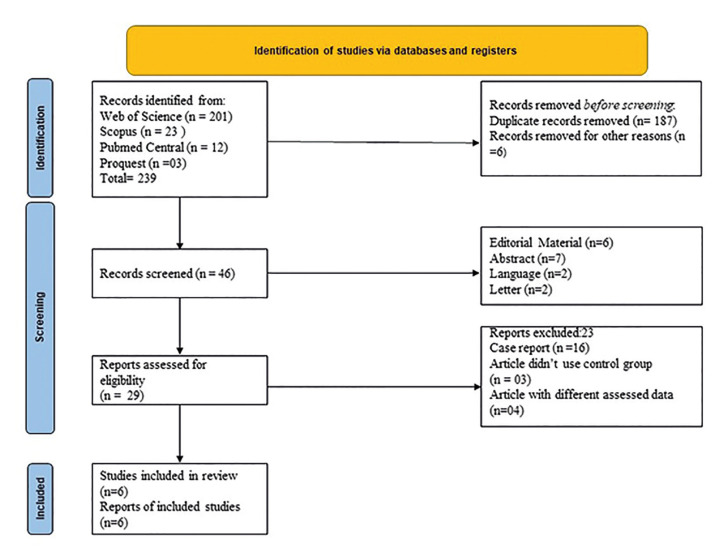
Flowchart of the studies selected for review and analysis.

A review of the literature identified 239 potential, relevant articles; ultimately, 6 m*et al*l criteria, which used for meta-analysis (Fig. 1). The six studies included 153 patients who underwent dental extraction. Their characteristics are shown in Table 1. Most of the studies were from Brazil, Belgium and Italy. The healing index 7 and 14 days after dental extraction and the VAS score were compared in three studies; two studies of different cohorts reported differences in the percentage of bone formation, socket width, and socket fill between the control and L-PRF groups.

**Figure 2:**
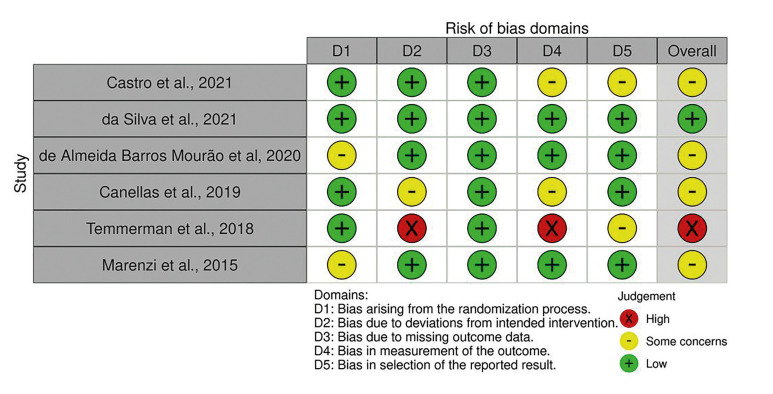
Risk of bias of included studies. Meta-analysis of socket width, socket filling and bone formation in control and L-PRF treated dental extraction patients (split mouth study).

- Quality assessment

was used to quality assessment of the included RCTs were used Cochrane Collaboration’s tool to assess risk of bias. The observation suggest that one study had high risk of bias; One trial had a low risk of bias; and four trials an unclear risk of bias (Fig. 2).

- Meta-Analysis for socket width, socket filling and bone formation in control and L-PRF treated dental extraction (split mouth study)

**Figure 3:**
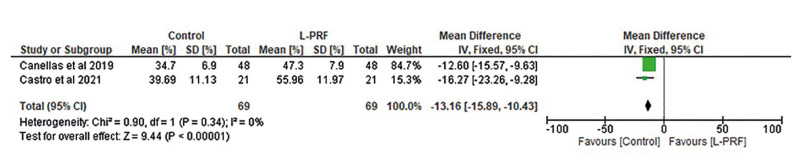
Forest plot of studies reporting the percentage of bone formation in control and L-PRF treated dental extraction patients.

The percentage of bone formation after dental extraction in control and L-PRF-treated patients was compared in two studies. The results of the statistical analysis are shown in Fig. 3. L-PRF group indicates higher in bone formation than in controls, with a mean difference of -13.16 (-15.89, -10.43). The difference in heterogeneity between studies was significant (df=1; *p*<0.00001).

**Figure 4:**
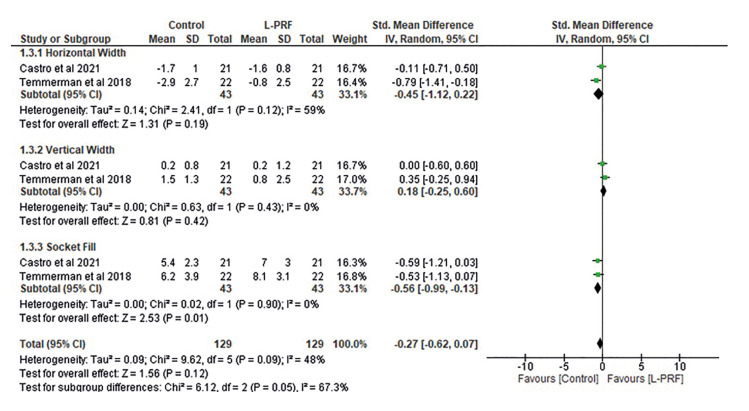
Forest plot of studies comparing socket width in a subgroup analysis of horizontal width, vertical width, and socket filling.

Fig. 4 shows the socket width based on a subgroup meta-analysis of the horizontal and vertical width and socket filling. The horizontal width was lower in controls than in the L-PRF group but the mean difference was small (-0.45 [-1.12, 0.22]) and the heterogeneity of the studies was moderate (I2 =59%). However, the vertical width was lower in treated patients than in controls, with a mean difference of 0.18 (-0.25, 0.6). The difference in heterogeneity was not significant (df=1; *p*=0.43; I2=0%). Conversely, socket filling was higher in the L-PRF group. The mean difference was -0.56 (-0.99, -0.13) and the difference in heterogeneity was not significant (df=1; *p*=0.90; I2=0%). Overall, the mean difference between groups in the subgroup analysis was low (-0.27 [-0.62, 0.07]) and the difference in heterogeneity was moderate (I2=48%), but not significant (*p*=0.09).

- Meta-analysis of the healing index

The healing index was compared between groups in a subgroup analysis of days 7 and 14 after dental extraction based on three different studies (Supplement 1). The index was higher in the L-PRF group than in controls on both post-operative day 7 and post-operative day 14. Overall, the index declined and the difference in heterogeneity was significant.

- Meta-analysis of VAS score

The VAS score was used to determine the pain level on day 7 after surgery (Supplement 2), and was lower in treated patients. There was lower VAS observed in L-PRF implant treated dental extraction than control group. Mean difference was observed higher upto 1.26 [1.00, 1.51] and significant difference was observed in the heterogenicity between them (df = 1; *p*< 0.00001).

## Discussion

Dental extraction is the most common form of oral surgery. Patients who undergo dental extraction experience discomfort for several days post-operatively due to swelling and pain in the vicinity of the surgical site (18). Bone loss during dental extraction also contributes to the discomfort, as the socket heals slowly. Implants to prevent ridge formation and bone loss and thereby promote healing and reduce discomfort have been proposed. L-PRF implants are rich in proteins and growth factors that promote wound healing (19), reduce postoperative pain and improve epithelialisation (20). L-PRF treatment may therefore improve the quality of life in early post-operative period of dental surgery patients. However, a recent study showed that it does not significantly improve pain and wound healing after dental extraction (8). We therefore conducted a meta-analysis to determine the effect of L-PRF on dental extraction.

Data from six studies were analysed to assess the factors that contribute to ridge prevention and wound healing. The respective studies were RCTs with a split-mouth design. L-PRF may play a role in soft-tissue regeneration by stimulating collagen synthesis and fibroblast proliferation (21). Clotting is an important process during surgery because it accelerates homeostasis. L-PRF contains concentrated thrombin and fibrinogen, and the complex trimolecular structure of the fibrin matrix resembles the natural tissue matrix but with higher elasticity and resistance (22). These features contribute to improved socket filling after dental extraction. The results of study indicated higher percentage of bone formation in the L-PRF group than controls. Socket width was also analysed, by determining the differences in the horizontal and vertical width, together with socket filling. Better results were obtained in the treated group. A metanalysis study on plasma concentrate (PC) like L-PRF and P-PRF has shown that spontaneous healing and alveolar ridges formation improved, however no differences observed among L-PRF and P-PRF (23). Data of our investigation supported by study of Caponio *et al* (23), difference between these studies, as it focus on L-PRF and P-PRF treatment on bone formation and our report majorly involve the assessment of L-PRF on new bone formation.

Post-operative pain and delayed wound healing contribute to the discomfort experienced by dental extraction patients, thus highlighting the importance of ridge prevention (24). L-PRF has been shown to relieve pain after tooth extraction (25). By sealing the socket with a membrane, L-PRF prevents the entry of debris and food and thus reduces pain stimuli (26). It also stimulates host defence mechanisms, by regulating the immune system, thus enhancing healing and relieving pain, in addition to warding off infection. These observations were supported by our meta-analysis, which showed a significantly higher healing index and better pain reduction in the L-PRF group.

Some limitations should be mentioned. First, only six publications were analysed and the patients were assessed for only 14 days. Also, many of the included studies had a moderate risk of bias. In addition, none of the studies assessed all parameters of interest.

## Conclusions

In conclusion, our meta-analysis showed that the L-PRF implant prevents ridge formation by improving both the percentage of bone formation and socket width (improved horizontal width and socket filling). Moreover, it improves the healing index and reduces the VAS score significantly.

## Figures and Tables

**Table 1:** Characteristics of the studies comparing socket width, pain index (VAS) and Healing Index in Control and L-PRF treated dental implants included in the meta-analysis.

Author, Year	Country	Study Design	Sample size	Socket width (mm)		Bone Formation (%)		VAS		Healing Index		Age	Gender (M/F)	Conclusion	Reference
				Control	L-PRF	Control	L-PRF	Control	L-PRF	Control	L-PRF				
Castro et al., 2021	Belgium	Randomized controlled clinical split-mouth trial	21	Socket filling= 5.4±2.3 Horizontal: -1.7±1.0 Vertical: 0.2±0.8	Socket filling = 7.0±3.0 Horizontal: -1.6±0.8 Vertical: 0.2±1.2	39.69± 11.13	55.96± 11.97	--	--	--	--	64.4± 12	6/15	No affect dimension, Superior socket healing Superior bone formation	(12)
Canellas et al., 2019	Brazil	Single blind Randomized controlled clinical	48	--	--	34.7± 6.9	47.3± 7.9	--	--	--	--	44.8± 26	21/27	L-PRF should be always considered	(13)
Marenzi et al., 2015	Italy	Randomized clinical trial	26	--	--	--	--	4.5± 0.7	3.2± 0.3	7 Day: 4.9± 0.3	7 Day: 4.5± 0.5	53±4	9/17	Use of L-PRF in post extraction sockets filling is efficient and useful procedure	(14)
										14 Day: 4.3± 0.3	14 Day: 4.2± 0.2				
da Silva et al., 2021	Brazil	Randomize, double blinded, split mouth study	20	--	--	--	--	0.37± 0.17	0.00± 0.0	7 Day: 3.64± 0.15	7 Day: 4.64± 0.14	23± 3.28	6/14	Use of L-PRF improves soft tissue healing process and decrease post operative pain	(15)
										14Day: 4.48± 0.13	14Day: 4.9± 0.07				
de Almeida Barros Mourão et al, 2020	Brazil	Randomized, double blinded, split mouth study	16	--	--	--	--	5.12± 1.08	4± 1.15	7 Day: 3.18± 0.54	7 Day: 3.81± 0.65	Control 38.1± 10.5 L-PRF 36.5± 11.4	Control 9/7 L-PRF 10/6	--	(16)
										14Day: 4.5± 0.51	14Day: 4.75± 0.44				
Temmerman et al., 2018	--	Randomized, double blinded, split mouth study	22	Socket filling = 6.2±3.9 Horizontal: -2.9±2.7 Vertical: 1.5±1.3	Socket filling = 8.1±3.1 Horizontal: -0.8±2.5 Vertical: 0.5±2.3	--	--	--	--	--	--	54±11	15/7	--	(17)

## Data Availability

All data generated or analysed during this study are included in this article. Further enquiries can be directed to the Correspondence.
